# Integrative review on *Acinetobacter baumannii* as a multidrug-resistant pathogen: resistance mechanisms and therapeutic perspectives in the context of nosocomial infections

**DOI:** 10.1590/0037-8682-0268-2025

**Published:** 2025-11-17

**Authors:** Pedro Henrique Melo Lima, Caio Ferraz Lopes, João Pedro Camargo Freire, Lucas Dias Feliciano, Rebeca Cristina Oliveira Amorim, Mateus Lima Mota, Filipe França Cirqueira, Fabian Fellipe Bueno Lemos, Silvia Helena Sousa Pietra Pedroso, Fabrício Freire de Melo

**Affiliations:** 1 Universidade Federal da Bahia, Instituto Multidisciplinar em Saúde, Vitória da Conquista, BA, Brasil. Universidade Federal da Bahia Instituto Multidisciplinar em Saúde Vitória da Conquista BA Brasil; 2 Universidade Federal de Pernambuco, Departamento de Antibióticos, Recife, PE, Brasil. Universidade Federal de Pernambuco Departamento de Antibióticos Recife PE Brasil

**Keywords:** Antimicrobial resistance, Epidemiology, Healthcare-associated Infection, Treatments

## Abstract

*Acinetobacter baumannii* is a gram-negative opportunistic pathogen associated with high morbidity and mortality in nosocomial infections, particularly in intensive care units. Multidrug resistance (MDR) is mediated by efflux pumps, lipopolysaccharide modifications, aminoglycoside adenylyltransferases, FosA, structural alterations, and production of enzymes that inactivate antibiotics, such as carbapenemases. These factors limit the therapeutic options and increase clinical challenges, as there are currently few drugs or combinations with therapeutic success against *A. baumannii* infections. Some strategies and new drugs, such as cefiderocol, eravacycline, sulbactam-darlobactam, tigecycline, and their combinations with colistin, are being tested and have shown apparent advances. This integrative review discusses the current resistance mechanisms and emerging therapeutic strategies aimed at overcoming the growing threat posed by MDR *A. baumannii*.

## INTRODUCTION

*Acinetobacter baumannii* (*A. baumannii*) is a bacterium of great clinical relevance today due to its spread in the hospital environment and, consequently, its relationship with opportunistic nosocomial infections and is one of the major concerns regarding current infections due to its high fatality rate^1^. In many cases, this bacterium is related to pneumonia, especially in association with mechanical ventilation; urinary infection, typically associated with bladder catheters placed for a long time; and meningitis, especially in patients after neurosurgery^2^^,^^3^.

The current major concern is the widespread resistance of *A. baumannii* to various classes of antibiotics used in the clinical management of its infections, which reduces the chances of therapeutic success and increases the likelihood of death^3^. The World Health Organization (WHO) has listed carbapenem-resistant *A. baumannii* (CRAB) as one of the world's critical priority bacteria^4^ and ratified this warning in 2024, with permanence within this category on the new list^5^.

Understanding the mechanisms involved in this resistance is essential for establishing new treatment alternatives and developing drugs that avoid these mechanisms to achieve greater success in containing these infections.

This review aimed to provide a comprehensive overview of the epidemiology, resistance mechanisms, and current therapeutic strategies targeting MDR *A. baumannii* in the context of increasing nosocomial infections.

## METHODS

This integrative review was conducted by searching PubMed/MEDLINE, Embase, Web of Science, Scopus, and the Cochrane Library to identify articles that could answer the guiding question: “what are the resistance mechanisms and therapeutic strategies currently available for multidrug-resistant *Acinetobacter baumannii* in nosocomial infections?”. The following search string was applied: ("*Acinetobacter baumannii*" OR "*A. baumannii*" OR "MDR *A. baumannii*" OR "CRAB") AND ("resistance" OR "resistance mechanisms" OR "antimicrobial resistance" OR "multidrug resistance" OR "carbapenemase" OR "efflux pumps" OR "lipopolysaccharide modification" OR "genetic mutations") AND ("nosocomial infection" OR "healthcare-associated infection" OR "hospital infection" OR "intensive care unit" OR "ICU") AND ("treatment" OR "therapeutics" OR "antibiotic therapy" OR "combination therapy"). Original studies and relevant reviews were included without initial language restrictions, but articles published in English were prioritized. We conducted a qualitative synthesis of the findings, highlighting the methodological limitations and knowledge gaps.

## RESISTANCE EPIDEMIOLOGY

Infection records in intensive care units (ICUs) indicate the frequency of *A. baumannii* outbreaks in several continents, mainly in Europe and North and South America^6^. These outbreaks were mainly caused by three MDR clones, although new strains are now common in some parts of the world, with reported cases in the United States, Canada, South America, Europe, Africa, the Middle East, Southeast Asia, and Australia^7^.

In a global assessment of the activity of antimicrobials against MDR gram-negative pathogens, *A. baumannii* accounted for 2-10% of all infections, with resistance rates increasing in 2017, showing a global increase of >40% in resistance between 2004 and 2014. When analyzed by continental region, the overall MDR among *A. baumannii* was the lowest in North America (31%), whereas >50% of *A. baumannii* isolates collected in Africa, the Middle East, and Latin America were MDR. In addition, the highest overall rates of MDR were observed among *A. baumannii* isolates, 44% of which were MDR^8^.

According to the Study for Monitoring Antimicrobial Resistance Trends (SMART), conducted with strains isolated from 48 countries between 2011 and 2014, *A. baumannii* had lower MDR rates in North America (47%) and higher rates in Europe and the Middle East (>93%), with higher rates in ICUs. Antimicrobial susceptibility profiles varied by region, with no tested antimicrobial agent able to inhibit >70% of those infected with *A. baumannii*^9^. Recent studies, in line with data presented in the SMART report, have indicated the prevalence of *Acinetobacter* MDR infections in the Gulf Cooperation Council, Middle East, and North Africa (MENA) regions^10^. For example, alarming levels of resistance to *Acinetobacter* MDR bacteria have been reported in ICUs in Saudi Arabia, Qatar, Kuwait, and the United Arab Emirates^11^^-^^13^.

Studies carried out by the SENTRY Antimicrobial Surveillance Program based on *Acinetobacter spp.* showed the percentages of susceptibility to meropenem in some regions of the globe, including Asia/Pacific (21.0%), Europe (22.2%), Latin America (13.7%), and North America (54.4%). Among the *Acinetobacter spp.* samples collected by Seifert et al.^14^ between 2016 and 2018, the percentages of susceptibility to meropenem were as follows: Africa/Middle East (17.2%), Asia/South Pacific (31.4%), Europe (33.8%), Latin America (19.6%), North America (63.6%), and globally (32.9%).

In South Korea, only 22.4% of the samples isolated during the prepandemic period (2015-2020) were susceptible to carbapenems^15^. In a Chinese hospital study conducted during the COVID-19 pandemic, 60% of the collected pathogen samples were resistant to carbapenems^16^. Another important factor during this period was the increase in nosocomial infections caused by carbapenem-resistant *A. baumannii* in ICU patients, especially in Italy and the USA^17^^-^^19^.

Tests have identified colistin-resistant strains in several countries, such as Spain, where a study revealed 19.1% resistance^20^, and a recent multicenter study carried out in southern Europe revealed that resistance was as high as 47.7%^21^. In South Korea, surveys have reported a resistance rate of 30.6 %^22^.

## RESISTANCE MECHANISMS

The main resistance mechanisms of *A. baumannii* and their descriptions are summarized in a table (Table 1).

**TABLE 1:** Main resistance mechanisms of *A. baumannii.*

RESISTANCE MECHANISMS	DESCRIPTION	RELATED GENE, PROTEIN, OR ENZYME
Efflux pumps	Transport antibiotics from the intracellular to the extracellular environment	Five different transporter protein families: SMR, DMT, ABC, RND, and MATE
Carbapenemases	Enzymes capable of hydrolyzing and therefore inactivating beta-lactam antibiotics	Mainly OXA β-lactamases, encoded by genes as *bla*_*OXA-23-like*_ *, bla*_*OXA24/40-like*_ and *bla*_*OXA-51-like*_ . But other enzymes such as New Delhi metallo-β-lactamase-1 encoded by the *bla*_*NDM-1*_ gene can also be found
Change in the structure of LPS	Production of cationic groups that bind to lipid A in order to reduce its electronegative character; complete loss of LPS; addition of pEtN to the structure of lipid A by a phosphoethanolami	Overexpression of the pmrCAB chromosomal operon; mutations in the *lpxA*, *lpxC,* or *lpxD* genes; plasmid gene mcr-1
Fluoroquinolones resistence	Alteration in the structure of type II topoisomerases and protection of binding site	Mutations in *gyrA* and *parC* (e.g. Ser83Leu, Ser80Leu), qnr proteins, AAC(6')-Ib
Antifolate resistance	Resistance via alternative enzyme variants and efflux	*sulI, sulII, dfrA1, dfrA12, dfrA14*; efflux pumps such as OqxAB
Fosfomycin resistance	Inactivation of drug, efflux, and changes in membrane permeability	*fosA, abaF*, *abpr* genes
Aminoglycosides heteroresistance	Gene amplification and enzymatic modification of aminoglycosides	*aadB(ant2")Ia, aph(3')-Via, AphA1 (Tn6020), RecA*
Amphenicols resistance	Drug efflux and enzymatic acetylation	*catA1, catA2, catB11, catB3, catB8; cmlA5, craA, abrp*
Tetracyclines resistance	Efflux pumps and enzymatic inactivation (TetX variants)	tet(A), tet(X3), tet(X4), tet(X6); adeABC, adeIJK, adeFGH

## EFFLUX PUMPS (GENERAL OVERVIEW)

Some strains of *A. baumannii* possess many of these transporters,^23^ which “expel” antibiotics and reduce their intracellular concentrations ​^24^. Efflux pumps are of particular concern because of their role in resistance to various biocides, including chlorhexidine. *Acinetobacter* chlorhexidine efflux protein, found in *A. baumannii*, expels the biocide antiseptic and survives in hostile environments^25^^,^^26^. 

Efflux pumps are associated with resistance to antibiotics such as macrolides, tetracyclines, and quinolones^27^. Different mechanisms are associated with efflux pumps, which are divided into different families^24^^,^^28^.

## SMALL MDR (SMR) - EFFLUX PUMPS

SMR are a family of four functional subdivisions that transport the small, charged metabolite guanidinium, bulky hydrophobic drugs, antiseptics, polyamines, and glycolipids^29^. Guanidinium exporters are the most common microorganisms^30^. 

## MAJOR FACILITATOR SUPERFAMILY (MFS) - EFFLUX PUMPS

MFS promotes substrate translocation by exploiting the free energy stored in the ion or solute gradients generated by primary transporters^30^. The MFS proteins transport various substrates^31^. In *A. baumannii* there are three MFS transporters are important for MDR: CraA, which provides resistance to chloramphenicol; AmvA, which provides resistance to erythromycin; and AbaQ, which transports quinolones^32^^,^^33^.

## RESISTANCE-NODULATION DIVISION FAMILY (RND) - EFFLUX PUMPS

RND in *A. baumannii* can expel most antibiotics among other substrates^34^. Some of its proteins that confer resistance are AdeABC, which contributes to resistance to aminoglycosides, trimethoprim, chloramphenicol, fluoroquinolones, and tetracyclines; AdeFGH, which confers resistance to trimethoprim, tetracycline, clindamycin, and fluoroquinolones; AdeIJK, which acts on β-lactams, chloramphenicol, tetracycline, erythromycin, lincosamides, fluoroquinolones, fusidic acid, novobiocin, rifampin, and trimethoprim; AbeD, which confers protection against ceftriaxone, gentamicin, rifampin, tobramycin, and benzalkonium chloride; and ArpAB, which ensures resistance to amikacin and tobramycin^32^^,^^35^.

## CARBAPENEMASES AS A ROUTE OF RESISTANCE TO CARBAPENEMS

Carbapenemases are enzymes capable of hydrolyzing and inactivating antibiotics of β-lactam antibiotics. β-lactamases are classified according to the classification scheme based on amino acid sequences, with class D being the most evident in MDR *A. baumannii* strains, whereas class B is seen less frequently^36^^,^^37^. This predominance of class D is probably related to their ability to integrate efficiently into the genome of local strains, their dissemination by plasmids, and their stability, which facilitates their maintenance and propagation. The lower prevalence of class B genes may be related to their even more restricted dissemination and regional factors affecting the circulation of resistance genes^38^.

Oxacillinase (OXA) β-lactamases are enzymes belonging to class D and are encoded by groups of genes, such as *bla*_*OXA-23-like*_ , *bla*_*OXA24/40-like*_ and *bla*_*OXA-51-like*_^39^. One study showed that increased expression of OXA-23, the first carbapenemase identified in *A. baumannii* and encoded by *bla*_OXA-23_^39^^,^^40^, increased the minimum inhibitory concentration (MICs) of imipenem by 128 times^41^. The ability of this enzyme to change its conformation, as proven in recent studies^42^, suggests that this mechanism is intrinsically related to the broad spectrum of β-lactams with compromised therapeutic action. OXA-24/40-like is originated from the *bla*_*OXA24/40-like*_ gene group and strains containing this gene showed higher MIC values than those with *bla*_*OXA-23-like*_ , even though the number of OXA-24/40 β-lactamases is more restricted than the OXA-23 group^39^^,^^40^^,^^43^. 

New Delhi metallo-β-lactamase-1 (NDM-1) is a class B β-lactamase synthesized from the *bla*_*NDM-1*_ of *A. baumannii* strains in infections in the city of New Delhi^44^. It can successfully hydrolyze all members of the β-lactam family, except monobactams, is not affected by β-lactamase inhibitors, and provides resistance to other antibiotics outside this class owing to its alternative mechanisms of action^44^^-^^46^.

## CHANGES IN THE STRUCTURE OF LIPOPOLYSACCHARIDE

*A. baumannii* already has resistance mechanisms to polymyxins based on alterations in the structure of the lipopolysaccharide or the complete loss of this molecule from its outer membrane^47^^-^^49^. Several factors are involved in this resistance, such as genetic mutations, reduced production of cofactors for LPS production, and lack of stabilizing proteins^50^^,^^51^.

Overexpression of the *pmrCAB* chromosomal operon is one of the main factors involved in the resistance of *A. baumannii* to colistin, once the influence of the *pmrA* and *pmrB* genes on the structure of LPS has been discovered^52^^,^^53^. Their mechanism involves inducing the production of cationic groups attached to lipid A to reduce its affinity for polymyxins^52^^,^^54^. The PmrB protein phosphorylates and activates PmrA, which in turn induces the expression of *pmrC*, which encodes the phosphoethanolamine transferase PmrC, to add the phosphoethanolamine (pEtN) group to lipid A^55^^-^^57^.

Mutations in *lpxA*, *lpxC*, or *lpxD* genes prevent lipid A synthesis. The loss of one of the main sites of action of polymyxins increases the MIC, making their use for the treatment of this type of strain unfeasible^58^. However, one study indicated that *A. baumannii* with this mutation is more susceptible to immune system defenses promoted by neutrophils^59^.

As for plasmid interference, mrc-1, despite recent evidence of its possible relevance to polymyxin resistance in *A. baumannii*^54^, is a gene capable of encoding a phosphoethanolamine transferase that adds pEtN to the structure of lipid A^56^.

## RESISTANCE TO FLUOROQUINOLONES

Changes in the amino acid sequence, and consequently, changes in the interaction of type II topoisomerases with fluoroquinolones, are generally characterized as recurring mechanisms in bacteria for resistance to these antibiotics^60^. The most recurrent mutations occur in the A subunit of DNA gyrase and the C subunit of topoisomerase, which are expressed by *gyr*A and *par*C, respectively. Studies have shown that alterations in serine amino acids are the most significant and recurrent for resistance, such as the Ser83Leu mutations in the *gyr*A genes and Ser80Leu in *par*C^61^^,^^62^. Unlike other bacteria, *A. baumannii* shows high resistance, even to a single double mutation involving at least one change in each of the subunits mentioned^61^.

Proteins that protect the sites of type II topoisomerase enzymes and DNA are synthesized by the *qrn* plasmid gene family^63^. These proteins destabilize the quinolone-binding site with the enzyme through new interactions, enabling regions to form again during distension and maintaining the activity of type II topoisomerases^64^^-^^66^.

The expression of efflux pumps that resist the action of fluoroquinolones in *A. baumannii* is mainly related to the QepA and OqxAB pumps^67^^,^^68^. However, other studies have reported discrepant results, with little influence or expression of these pumps on *A. baumannii* resistance^69^^,^^70^.

## RESISTANCE TO ANTIFOLATE ANTIBIOTICS

Different mechanisms are involved in the resistance of these classes; however, as they are easily transferable, they are commonly related to each other^71^. The *sul I* and *sul II genes* encode dihydropteroate synthase enzymes with structural alterations that decrease their affinity for sulfonamides, but maintain their interaction with p-aminobenzoic acid, a precursor substrate for folic acid in this metabolic pathway^72^. The *sul I* gene is generally associated with transposons, which delimit resistance to other microbes^72^^,^^73^.

Genes from the dfr family, such as *dfrA1*, are responsible for producing dihydrofolate reductase with a structure altered enough to maintain interaction with the substrate dihydrofolate, but drastically lose interaction with trimethoprim, which increases the MIC in strains carrying these genes^74^.

The expression of efflux pumps may also be related to the mechanism of antifolate resistance in *A. baumannii*, as OqxAB has been shown to confer resistance to various classes of antibiotics, including trimethropine^75^.

## FOSFOMYCIN RESISTANCE

FosA is a glutathione S-transferase synthesized by a gene of the *fosA* family that inactivates fosfomycin by altering its MurA-binding structural portion, which is the site of action of this drug, and is widespread in *A. baumannii*^76^^,^^77^.

The AbaF efflux pump, encoded by the *aba*F gene, is associated with loss of susceptibility to fosfomycin, as its nonexpression resulted in an eight-fold increase in the therapeutic efficiency of this drug in *A. baumannii* strains^78^. These pumps appear to confer additional resistance and virulence traits, as the expression of other efflux proteins did not show the same results, whereas AbaF, in addition to reducing susceptibility, favored the formation of biofilms^78^.

The *abpr* gene has been shown to reduce susceptibility to fosfomycin and other classes of antibiotics, owing to changes in the selection characteristics of cell membrane permeability, and consequently, the therapeutic efficiency of the drugs^79^.

## AMINOGLYCOSIDES HETERORESISTENCE

As observed via hybridization, 82.7% of the bacterial strains of *A. baumannii* that show resistance to aminoglycosides have the *aph(3')-Via* gene, however, this resistance is multifactorial, and is related to more than one gene or mechanism^80^.

The basis of heteroresistance to aminoglycosides is the amplification of the *aadB(ant2″)Ia* gene, which encodes an aminoglycoside adenylyltransferase, capable of adding adenosine monophosphate to other molecules, making the strain capable of inactivating tobramycin and gentamicin. This gene is highly amplified in a RecA-dependent manner and is responsible for genetic recombination, gene expression regulation, and DNA repair^81^. This amplification occurred stochastically, even without antibiotic pressure, in approximately 1 in 200 cells​^82^.

Another gene related to aminoglycoside resistance is *aphA1,* which encodes an enzyme called phosphotransferase that modifies aminoglycosides by adding a phosphate group to them^24^. However, when the number of gene copies is very high (>40), the bacterial response is modified and loses its ability, which suggests that a high number of copies overload the cell^83^. 

## AMPHENICOLS RESISTANCE

The genes that confer resistance to amphenicols are redundant, more than one for the same antibiotic, which increases resistance, one of which is the *catB8* gene that allows the expression of CatB8, which has a greater capacity to process and neutralize the effect of the antibiotic because this protein can bind to up to four molecules of chloramphenicol^84^^,^^85^. 

Some strains of *A. baumannii* harbored the *abrp* gene, which, when deleted from the bacterial gene, showed reduced susceptibility to chloramphenicol (>8-fold)^86^. Other genes, such as *craA* and *cmlA5* have been identified as responsible for resistance to chloramphenicol and other antibiotics in different strains of bacteria. In addition, impermeability of the outer membrane contributes to amphenicol resistance in *A. baumannii*^85^.

Another gene of significant importance in chloramphenicol resistance is ABUW_0982, which, when deleted via mutations in the transposon, increases bacterial susceptibility to this antibiotic^87^.

## TETRACYCLINE RESISTANCE

The first antimicrobial drug efflux pumps to be recognized were the Tet(A) pumps, which belong to the MFS, that remove the drug from the cytoplasm to the periplasm in *A. baumannii* and; subsequently, the RND-type transporters AdeABC and AdeIJK extrude tigecycline through the outer membrane^88^^-^^92^. The *tet(X3)* and *tet(X4)* genes found in some strains of *A. baumannii* significantly increase the minimum therapeutic dose of tetracyclines together with the superexpression of RND efflux pumps, which are the main resistance mechanisms to tigecycline^93^^,^^94^. TetX modifies first- and second-generation tetracyclines, with tigecycline as one of its substrates^95^.

The main mechanisms of resistance to tetracyclines involve efflux pumps, most commonly AdeABC and AdeFGH, and in some cases, AdeIJK^96^. Among these, the most studied efflux pump is AdeABC, whose activity is regulated by a set of genes called AdeRS. Mutations in these genes can cause deregulation and overexpression of the AdeABC efflux pump, thereby reducing the susceptibility of the pathogen to tetracyclines^97^^,^^98^.

## CLINICAL MANAGEMENT

The main therapeutic and clinical management strategies are summarized in Table 2.

**TABLE 2:** Therapeutic alternatives in clinical management.

DRUGS/COMBINATIONS	CLASS	ACTIONS MECHANISMS	OBSERVATIONS
Sulbactam-durlobactam	β-lactamase inhibitors	They compete with and irreversibly inhibit β-lactamase, lowering the minimum inhibitory concentration	Durlobactam is a state-of-the-art inhibitor with broad action against β-lactamases of Ambler classes A, C and D that is being developed with sulbactam for the treatment of MDR *A. baumannii*
Ampicillin-sulbactam with meropenem and polymyxin B	Ampicillin and meropenem: β-lactam Polymyxin B: polymyxin	Ampicillin and meropenem prevent transpeptidation of the cell wall; polymyxin B is a cationic detergent of the outer membrane	This association demonstrated quick elimination of *A. baumannii*, but isolated therapies or the combination of only two resulted in bacterial regrowth
Colistin	Polymyxin	Cationic detergent that deactivates the bacterial outer membrane	Associations with other drugs have shown a higher success rate, such as use with meropenem or a tetracycline plus ampicillin-sulbactam.
Eravacycline	Tetracycline	Exerts its antibacterial action by binding reversibly to the bacterial 30S ribosomal subunit	It is a new antibiotic introduced on the market that has shown high synergy with β-lactams to treat *A. baumannii*, especially when used together with ceftazidime.
Cefiderocol	Siderophore cephalosporin	Inhibits cell wall synthesis by binding to PBPs and β-lactamases; enters via active iron transporters	Effective against CRAB and MBL strains; recommended for combination therapy
Tigecycline	Glycylcycline	Binds to 30S ribosomal subunit with high affinity, blocking protein synthesis	Resistance may occur via efflux pumps; synergy with β-lactams and polymyxins

## SULBACTAM-DURLOBACTAM

Sulbactam is a competitive inhibitor of serine β-lactamase, which binds to penicillin-binding proteins. Research has shown that sulbactam alone has direct antibacterial activity against clinical isolates of *A. baumannii*^99^. Several studies have suggested that high-dose sulbactam is advantageous for the treatment of MDR *A. baumanni*^100^^,^^101^; however, combination therapy is the most effective treatment regimen for these infections^102^^,^^103^. Durlobactam is a state-of-the-art β-lactamase inhibitor, with broad action against β-lactamases classes A, C and D. Durlobactam recovers the *in vitro* efficacy of sulbactam against strains of the *A. baumannii-A. calcoaceticus* (ABC) complex, for which sulbactam has low effectiveness. Durlobactam reduced sulbactam's minimum inhibitory concentration by 16-64 times^104^, so this combination is being developed for the treatment of infections caused by the ABC complex^105^ and for the treatment of infections caused by CRAB^106^. 

A randomized clinical trial evaluated the efficacy and safety of sulbactam-durlobactam compared with colistin, each combined with imipenem-cilastatin. The final study population consisted of 125 patients with confirmed CRAB infection. The 28-day fatality rate was lower in the sulbactam-durlobactam group than in the colistin group (19.0% vs. 32.3%), and the incidence of nephrotoxicity was lower (13% vs. 38%; P<0.001)^107^.

## AMPICILLIN-SULBACTAM WITH MEROPENEM AND POLYMYXIN B

In studies using the hollow fiber infection model, the administration of ampicillin-sulbactam at high doses (8/4 g every 8 h) combined with meropenem (2 g every 8 h) and polymyxin B (1.43 mg/kg body weight every 12 h with an initial loading dose) led to the rapid elimination of *A. baumannii* in 96 h, whereas isolated therapies and combinations of the two drugs resulted in bacterial regrowth^108^^,^^109^.

## ASSOCIATIONS WITH COLISTIN

A Greek study^110^ analyzed the treatment of patients with ventilator-associated pneumonia caused by *A. baumannii*. The patients were then treated with intravenous colistin, tigecycline, and ampicillin/sulbactam. Inhaled colistin was administered simultaneously. The result was 90% patient survival, which demonstrates the promising efficacy of the combination.

A Korean study^111^ found that patients with *A. baumannii* bacteremia who were treated with colistin and meropenem tended to have a higher clinical success rate than those treated with colistin monotherapy (61.3% vs. 40.0%).

## ERAVACYCLINE

Eravacycline and omadacycline, two new antibiotics, have shown promising results for the treatment of gram-negative infections^112^^,^^113^. Combination therapy, which is widely used in clinical practice^114^, has also proven to be effective with antibiotics already in use.

Eravacycline belongs to the tetracycline class of antibiotics owing to its similar chemical structure and mechanisms of action^115^^,^^116^. After *in vitro* tests based on the MICs, eravacycline was found to be highly effective against MDR strains^117^.

One study evaluated the activity of eravacycline combined with colistin against ten CRAB isolates and showed 10% synergy and no antagonism^118^. Tests that showed The most active combination against *A. baumannii* was eravacycline-ceftazidime and eravacycline-imipenem, which showed synergy against >50% of the isolates^119^. More recent studies have shown that the eravacycline-ceftazidime combination was the most potent against *A. baumannii* with 80% synergism^120^. 

## CEFIDEROCOL

Cefiderocol was designed for the treatment of carbapenem-resistant gram-negative pathogens, particularly nonfermenting species, such as *A. baumannii*^121^. The drug penetrates bacterial cells via active iron transporters, overcoming drug resistance caused by mutations in porin channels and overexpression of efflux pumps. It also has intrinsic stability against hydrolysis by carbapenemases such as KPC, OXA-23, OXA-48-like, OXA-51-like, and OXA-58^122^^-^^124^.

The clinical use of cefiderocol has been linked to higher fatality rates in certain groups of patients than the best available therapy^125^. Other studies have reported a high success rate for this molecule, especially in critically ill patients^126^^,^^127^. Thus, cefiderocol should be used with caution, particularly in individuals with complex clinical profiles, such as those with renal dysfunction or septic shock^128^.

## TIGECYCLINE

Compared to tetracyclines, tigecycline shows greater antibacterial potency owing to its higher binding affinity for the 70S ribosome, or more specifically, with helices 31 and 34 of the 16S rRNA at the head of the 30S subunit^129^^,^^130^. 

The treatment of choice for *A. baumannii* is based on clinical experience and in vitro susceptibility test^131^ s. A retrospective study of infections involving tigecycline treatment was conducted in the UK, isolating the cases of thirty-four patients and 68% of the isolates were susceptible to tigecycline. All patients were treated with a standard dosage regimen of 100 mg loading dose, followed by 50 mg of intravenous tigecycline every 12 h^132^. In a study conducted in Shandong, China, clinical isolates of *A. baumannii* were collected from patients in three hospitals. Overall, 1,026 strains of *A. baumannii* strains were identified. The rate of resistance to tigecycline was 13.4%, which was associated with the overexpression of efflux systems^133^. 

Emphasizing the effects of combinations, tigecycline have been reported in many studies and demonstrate that combination with β-lactams or polymyxin B can lead to high synergistic activity against MDR *A. baumannii*^118^^,^^134^^,^^135^.

## PRACTICAL RECOMMENDATIONS BASED ON THE REVIEWED DATA

Strengthen infection control measures in hospitals, particularly strict hand hygiene, isolation of colonized/infected patients, and environmental cleaning, to prevent *A. baumannii* dissemination.

Implementation of routine surveillance of antimicrobial resistance patterns in *A. baumannii* isolates to guide empirical therapy.

Prefer combination therapy (e.g., high-dose sulbactam with durlobactam, ampicillin-sulbactam plus meropenem, and/or polymyxin B) for MDR *A. baumannii* infections instead of monotherapy when supported by susceptibility data.

New agents (cefiderocol, eravacycline, and tigecycline) in critically ill patients when standard regimens fail or resistance is documented.

Reserve polymyxins and other last-line agents for documented infections to slow the development of resistance.

Encourage research on antibiotic to restore antimicrobial activity.

The development and application of hospital stewardship programs have focused on rational antibiotic use to reduce selective pressure.

## CONCLUDING REMARKS

*Acinetobacter baumannii* is a pathogen that is difficult to treat clinically because of its seemingly endless potential to acquire resistance to antibiotics and the wide dissemination of MDR strains. The main mechanisms of resistance in current clinical management include efflux pumps, the production of carbapenemases, and changes in LPS. In addition, it displays mutations in topoisomerases and expresses genes that inactivate antifolates, aminoglycosides, and fosfomycin. Presumably, effective treatment depends on combined therapeutic strategies because single therapies often fail or show limited results, making antibiotic combinations promising options.

Therefore, measures are required to prevent and control *A. baumannii* infections and elucidate their mechanisms of resistance. This includes the rational use of antibiotics, continuous monitoring of antimicrobial resistance, strict hospital hygiene measures, proper hand hygiene, and isolation of patients colonized or infected with MDR *A. baumannii*. Encouraging research into new therapies and combinations to develop new antibiotics that avoid the resistance mechanisms discussed and develop innovative therapeutic strategies is essential in this context and may offer hope. However, only judicious use of current treatments will slow the continued increase in resistance to this emerging pathogen.

## Data Availability

Research data is not available
